# Characterization of an Ancient Bimetallic Alloy from Moche Civilization (Peru)

**DOI:** 10.3390/ma16227211

**Published:** 2023-11-17

**Authors:** Marta Porcaro, Roberto Cesareo, Angel Bustamante, Antonio Brunetti

**Affiliations:** 1 Department of the Earth Sciences, University “La Sapienza” of Rome, 00185 Roma, Italy; 2 University of Sassari, 07100 Sassari, Italy; roberto.cesareo@gmail.com; 3 Solid State Physics Department, National University of San Marcos, Lima 15081, Peru; angelbd1@gmail.com; 4 Biomedical Sciences Department, University of Sassari, 07100 Sassari, Italy

**Keywords:** ED-XRF, Monte Carlo simulation, spectroscopy, Tumbaga

## Abstract

The Moche civilization in Peru developed marvelous metallurgy, primarily using alloys of gold, copper and silver, with the most famous of them called Tumbaga, which resembles pure gold after a depletion process on its surface. However, they also created objects with more standard single-layer alloys or gilding. To distinguish between these techniques in a non-destructive manner is essential. Here, we analyzed a thigh protector, composed of two parts, one seemingly in silver and the other seemingly in gold. The sample was analyzed using X-ray fluorescence measurements integrated with Monte Carlo simulation. The results show that the silver part is formed of a silver-based alloy covered in a corrosion layer, while the gold part is made of Tumbaga. Moreover, for the first time, the gold profiles of different Tumbaga gold objects, from the same burial, were compared, allowing us to obtain information about the standardization of their manufacture.

## 1. Introduction

The Moche civilization emerged approximately a thousand years before the Incan civilization, around the first century AD, and flourished until the seventh century in northern Peru [1,2,3]. During this period, it developed thriving architecture, characterized by numerous pyramids and an irrigation network that enabled them to overcome the arid environment. Probably the most notable part of their artistic production is linked to the production of ceramics, often finely decorated, depicting deities and anthropomorphic and zoomorphic figures, where the resemblance to real figures is remarkable. Many military figures and combat scenes are also represented, attesting to how important military activity was for this population. Alongside ceramic production, they also developed flourishing and distinctive metallurgy [2]. The same technological quality achieved in ceramics was at least matched, if not surpassed, by metallurgical production. The latter was particularly focused on the use of the triad of copper–silver–gold, materials used both separately and in alloy form, with varying percentages used to achieve the desired color of the artifact. It must be remembered that the Moon and the Sun were considered deities and were often represented through their colors [1,2]. In particular, the Moche civilization developed an alloy called Tumbaga, composed of a dominant amount of copper, typically ranging from 60% to 80%, and smaller quantities of gold and silver; however, visually, it resembled pure gold, and much more rarely, pure silver [4,5,6,7,8]. This effect was achieved through a chemical process, using some natural elements, which caused oxidation of the copper, which could then be removed from the surface. After removal, the remaining material exhibited strong porosity, which was reduced through subsequent processing (heating and hammering). This technique, in addition to the truly remarkable aesthetic aspect, allowed for the production of artifacts highly resistant to corrosion, to the extent that even today, they appear to be made of pure gold or an alloy where the amount of gold is dominant. The Moche themselves also used gilding, which, as is typical for ancient metal objects exposed to the environment, is almost always discovered considerably corroded, often losing a substantial part of its gold coating. The production of Tumbaga alloys, a name incidentally coined by Spanish conquistadors, is actually widespread in much of the Americas, particularly in Central America, such as in Colombia. Despite their uniqueness, spread and importance, Tumbaga artifacts have been relatively understudied. This is also due to the great difficulty encountered in studying these objects outside of a museum, effectively limiting the study to the use of portable instrumentation. Beyond its historical importance, the Tumbaga alloy is of great interest in modern technology, for example, in the production of electrodes [9,10].

**Figure 1:**
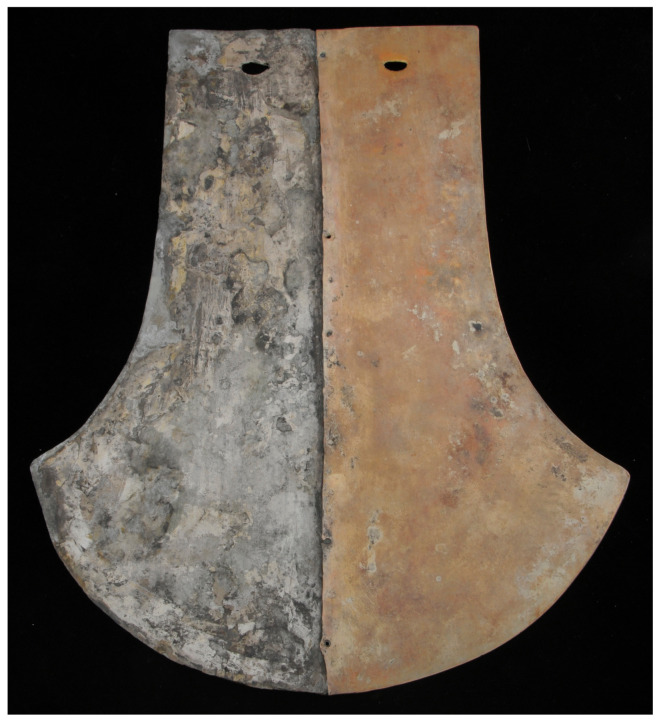
Bimetallic thigh protector. It is composed of two parts, one in silver color (**left side**) and one in golden color (**right side**), symbolizing the duality of Moon–Sun.

The sample studied in this work is a so-called thigh protector. It is divided vertically into two parts, one in a silver color and one in a golden color (see Figure 1), symbolizing the importance of the Moon–Sun symbology for this civilization.

This artifact is part of the treasure discovered in the Royal Tomb of Sipan, which is the burial site of high-ranking Moche dignitaries and leaders [1]. This tomb features a complex architecture, consisting of multiple levels totaling fourteen tombs, containing different generations of the ‘Lords of Sipan’, but generally just called the Lord of Sipan tomb. The mummies were adorned with incredibly valuable grave goods and offerings. The thigh protector comes from the funerary equipment of a mummy representing a warrior priest. It was the first object of its kind (two parts, gold–silver), previously only represented in many Moche images. Given the uniqueness of the piece, its characterization can provide valuable information about Moche metallurgy. It is currently housed in the Royal Museum of Sipan and, due to its rarity, cannot be sampled or taken out of the museum. For this reason, it was decided to employ a non-destructive technique based on portable instrumentation, i.e., X-ray Fluorescence (XRF or Energy-Dispersive XRF, EDXRF) spectrometry. XRF is a non-destructive technique capable of determining the elemental content of the analyzed sample. It is based on the interaction of X-ray radiation with matter.

For the range of energies used in this work, only three types of interactions can take place: photoelectric and scattering (both elastic and inelastic). One of the possible consequences of photoelectric interaction is the production of fluorescence photons. The energy of the fluorescence photons is characteristic of a specific chemical element, and thus, can be used for identification. Inelastic scattering will produce photons with energy lower than the photon that produced them, and they are not directly related to a specific chemical element. Elastic or Rayleigh interactions will not affect the energy of the photon that produced them and will not have a distinguishable or significant effect. All these phenomena are represented by a histogram, called a spectrum, showing the number of photons versus their energy. In addition to the peak contributions, which were explained earlier, and which aid in the identification and quantification of the chemical components of the examined sample, the background also plays an important role. This is primarily due to photon scattering and is not directly associated with a specific chemical element. However, it can be utilized to obtain information about “invisible” chemical components, which refers to undetectable fluorescence photons, specifically elements with a low atomic number. For example, the intensity of the background can be used to estimate the thickness of protective coatings used to shield the surface of ancient metals from corrosion (see reference [11]). These materials are composed of lightweight elements that do not produce measurable fluorescence photons with conventional XRF in air, but they alter the fluorescence photons emitted by the ‘heavy’ chemical elements of the metal. Of course, it is not possible to determine exactly the materials that make up the protective coating unless the chemical formula is known in advance. Only the average atomic number of it is known; however, this is sufficient to describe the behavior of the protective coating quite well.

The experimental measurements will also be simulated using the Monte Carlo technique, following a protocol that has already been applied in the analysis of various types of metals, including Tumbaga, yielding results significantly superior to those achievable with XRF alone [4]. The term ‘Monte Carlo method’ encompasses simulation techniques based on a probabilistic approach for problems of practically any nature that cannot be addressed via analytical approaches. Generally, problems with high dimensionality, meaning a very large number of parameters, fall into this category. Given this, it can be understood that there is an enormous number of Monte Carlo algorithms, each specialized for a specific class of problems. For the type of measurement conducted in this work, the Monte Carlo algorithm must be capable of simulating the interaction of X-ray radiation with matter, particularly predicting possible multiple interactions within the material, a phenomenon that is entirely unmanageable using analytical techniques, requiring complex multiple integrations. The protocol used will be described in detail in the next section.

## 2. Materials and Methods

The XRF system used here was developed at the University of Sassari. It is based on a silicon drift detector (1-2-3 SDD from Amptek, Bedford, MA, USA) and a rhodium transmission anode X-ray tube (Mini-X from Amptek, Bedford, MA, USA). The system allows for variable geometry, making it easily adaptable to the morphology of the surface to be analyzed. Having a flexible geometry is particularly important when analyzing cultural heritage because, in general, these are objects with complex morphology and, as a result, some parts are difficult to access using fixed-geometry instruments like those in commercial portable XRF systems. The typical setup involves placing the detector vertically about 2 cm from the surface, without any collimation or filter, and positioning the X-ray tube at an angle between 30 and 45 degrees relative to the detector and about 2 cm from the surface itself. The position of the detector, perpendicular to the surface, along with the relatively wide focal spot size, allows for the minimization of effects due to non-smooth surfaces, for example, with corrosion. In fact, envisioning a ‘rough’ surface, the collection of photons, especially those with low energy, would be affected not only by the internal structure of the sample but also by an irregular ‘peaks and valleys’ structure typical of non-smooth surfaces. To better explain this point, imagine a photon emerging from a ‘valley’ on the surface that, on its way to the detector, encounters the side of a ‘mountain’ it must pass through. This means that the attenuation experienced within the sample is further compounded by the transverse traversal of the ‘mountain’. Additionally, with the same composition, the attenuation will be higher for photons of lower energy, resulting in the potential non-uniform alteration of detected photons, and thus, introducing an error in the estimation of concentrations.

The tube operates at 40 kV with a variable current between 5 and 20 μA. The tube is collimated with a cylindrical collimator with a diameter of 1 mm, without any filter. The decision not to filter either the tube or the detector was made in order to utilize all the information present in the complete X-ray spectrum, primarily in the background, as described in the previous section.

The Monte Carlo simulation involves reproducing the measurement geometry, describing the emission from the X-ray tube, modeling the detector response and, finally, creating a sample model. The most critical part is characterizing the emission from the tube because it has a significant influence on the accuracy of the simulation.

However, characterizing the emission of a tube is not an easy task. In our case, we started by selecting the operating voltage to be used, which is 40 kV, at the minimum current because the latter does not significantly influence the shape of the emitted spectrum. Then, we positioned the detector that would be used in front of the tube output, but at a distance of about 1.2 m. Placing the detector far away serves to reduce the number of photons per second reaching the surface of the tube, which decreases with the square of the distance, as an excessive number of photons would increase the detector’s dead time, i.e., the time during which the detector is unable to record and distinguish photons, resulting in the loss of many potential counts and the incorrect identification of photons with the sum energy of two or more photons. An increase in dead time can also cause significant distortions in the shape of the peak, thereby impairing the system’s performance.

However, placing the detector far away means experiencing air attenuation in the tube–detector path. Attenuation can be mathematically corrected provided there is a sufficient number of photons reaching the detector, which does not occur for lower energies, such as in the case of the X-ray tube used here with a rhodium anode, for the L fluorescence lines emitted by the tube. In general, the absence, and therefore, the low likelihood of simulating low-energy X-ray tube emissions is not a major issue unless the concentrations of light elements, such as sulfur, or the fluorescence emissions of heavier elements at low energy, for example, the silver L lines when the K lines, are not available. In the measurements considered here, light elements or low-energy emissions are not important, except for the silver L lines. Thus, to obtain a well-characterized spectrum of the tube emission, an iterative procedure was adopted. Measurements were taken on a reference bronze sample under the same experimental conditions that would later be used for measurements on the Moche artifact. The measurement of the tube emission in air at a 1.2 m distance, corrected for attenuation introduced by the air itself, was used as the input for the Monte Carlo simulation of the experimental measurement. The spectrum obtained, for the above-mentioned problems, is not an accurate reproduction of the measurement. Therefore, the tube spectrum was iteratively corrected until a perfect simulation was achieved. When this happens, the spectrum of the tube obtained is a good representation of what is actually emitted. This long and complex process must be repeated whenever the tube’s operating voltage is changed. In theory, it is possible to take other approaches, such as simulating the operation of the tube itself based on data provided by the manufacturer, always using Monte Carlo. This approach would allow us to obtain emission spectra for each energy at the cost of simulation time (which is very long, in the order of a week). However, as mentioned before, it is conditioned by the availability of the technical data of the X-ray tube from the manufacturer, which are not often available. Currently, we are also exploring this path, but this approach was not used in this work.

The models for the X-ray tube emission, the geometry and the detector response will remain constant during the simulation. Only the sample model will vary depending on the quality of the simulated spectrum compared to the measured one. The initial model of the sample will primarily rely on observations, including the color and shape of the corrosion, as well as the fluorescence peaks in the measured spectrum. After this initial phase, an iterative cycle will be initiated based on the following steps:

(a) Monte Carlo simulation of the real experiment.
(b) Comparison of the simulated spectrum with the measured one.
(c) If differences are identified, in terms of the chi-square test or similar, the structure/composition of the sample will be modified, and the cycle will be repeated.

Using a traditional Monte Carlo code, i.e., a general-purpose one, even if created for the interaction of photons with matter, the execution of a single cycle would require from a few hours to several days of simulation, effectively rendering this approach impractical [12,13,14]. To address this type of issue, some fast Monte Carlo methods have been developed based on so-called variance-reduction techniques [15,16,17,18,19]. In this work, we utilized the code called *XRMC* [19]. It allows for simulating any type of X-ray experiment up to about 100 keV, from X-ray Fluorescence to phase-contrast tomography, to give two examples. In our case, simulating an XRF experiment with a spectrum quality similar, in a statistical sense, to the experimental one takes about 10–20 s on a standard laptop. XRMC leverages the *XRaylib* library as a database of atomic parameters involved in the simulation [20]. Initially developed at the European Synchrotron Radiation Facility (ESRF) in Grenoble, France, *XRaylib* is now constantly updated and used by a broad international community of users.

A separate discussion is required for the choice of the sample model, because both metal alloys composing the sample can be Tumbaga (silver and gold Tumbaga). The initial assumption for the sample composition starts from the sample observation. It is evident that the part that appears to be silver has a layer of corrosion, while the part that appears to be gold or gilded shows slightly darker areas, likely due to surface emergence and consequent copper corrosion, indicating an alloy containing copper. Based on these observations, various models were tested for the two alloys, ranging from a simple double layer (alloy plus corrosion or, in the case of gold, gilding) to seven-layer Tumbaga models simulating the typical Tumbaga concentration gradient [4]. As demonstrated in a previous study [4], Monte Carlo simulations are perfectly capable of distinguishing between gilding and Tumbaga, as well as between gradient models with different numbers or thicknesses of layers. When simulating a gradient, the thickness of individual layers is in the order of a micron or less and may vary from layer to layer. However, using a seven-layer model, each with its own thickness and the same components, in our case, gold, silver and copper, with relatively small variations in concentrations between the layers, means dealing with 28 parameters that need to be adjusted at each interaction. This means that, although each individual simulation lasts a short time, the total simulation time required to achieve the best fit can be lengthy. Unfortunately, these adjustments cannot be made automatically. This complexity might lead one to believe that there could be multiple solutions to the same problem, indicating what is known as an ill-posed problem. However, in reality, the sensitivity to parameter variations, even in such a complex structure, is very high. Therefore, it is relatively straightforward to differentiate between similar solutions. This explanation will be addressed in the upcoming sections, precisely determining the minimum concentration variations based on the layer being considered.

## 3. Results and Discussion

In this section, we report the results obtained using the XRF measurements and Monte Carlo simulations for the two parts of the object, separately. In both cases, several structures were considered, ranging from a single-layer alloy to a complex gradient-like structure formed of up to seven layers. Actually, after testing the 7-layer model, models with 9 and 11 layers were also tested, but no improvements were achieved. For this reason, they will not be discussed here.

### 3.1. Silver Side

**Figure 2:**
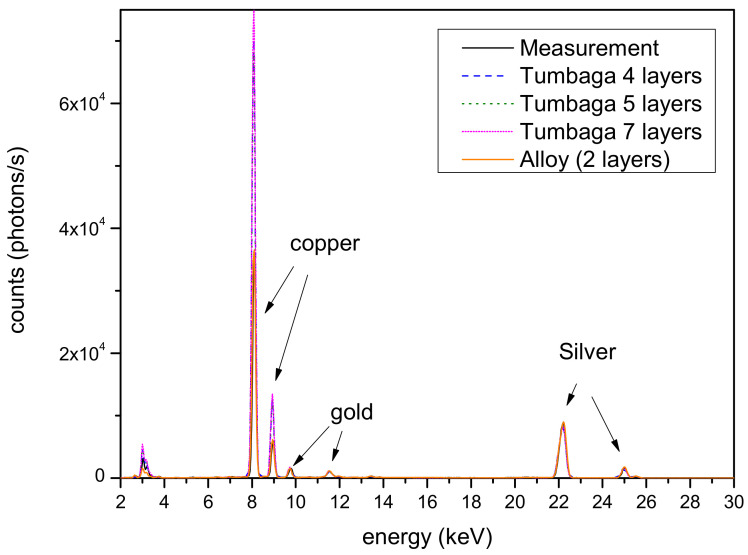
Thigh protector—silver area. Comparison of different models for the sample structure. To better highlight the quality of the fit, only the measurement and the simulation spectra with two layers are represented with a solid line.

**Figure 3:**
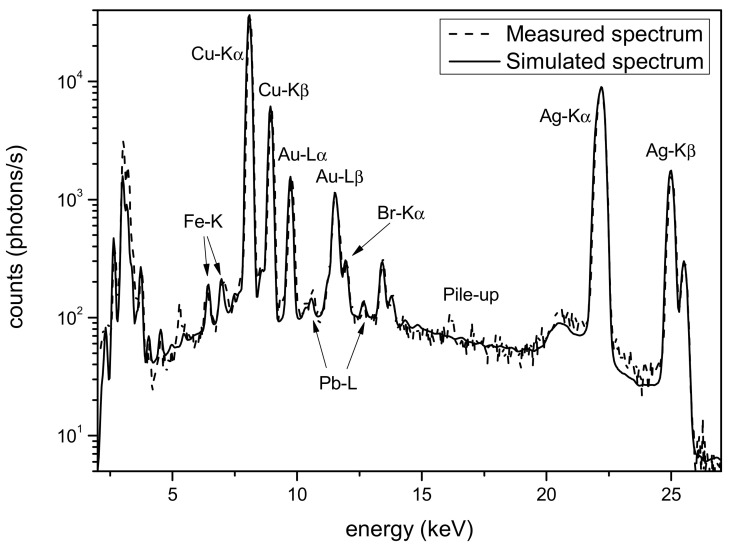
Thigh protector. Comparison of measured spectrum (dashed line) and simulated spectrum (solid line). The Monte Carlo simulation is based on a two-layer model of the sample.

The silver surface is apparently covered in certain areas by a layer of corrosion, partially removed during restoration [21,22,23]. For the Monte Carlo simulation of this part of the object, essentially, two classes of models were used: a double layer composed of a corrosion patina and a silver-based alloy, and a gradient model (silver Tumbaga) using models with four, five and seven layers. In Figure 2, a comparison of the best simulation for each of the models of the sample with different numbers of layers is reported, together with the real measurements. The figure clearly shows the different effects on the peak amplitudes of the various models, with the real measurement being well matched only by the two-layer model. Large differences in the low-energy part of the spectra, mainly in the K-line peaks of the copper, are clearly evident. The best model result from these simulations is shown in Figure 3 alongside the measured spectrum. It was obtained via the two-layer model, which is a corrosion patina covering a silver-based alloy. The composition of the two layers is reported in Table 1. The first layer is essentially composed of oxides; thus, the main components are oxygen, carbon and hydrogen. The actual contents of these elements are impossible to establish via Monte Carlo simulation, which determines a mean Z value. For this reason, in the first layer, as reported in Table 1, only the heavy and detectable elements, in terms of fluorescence signal, are reported.

### 3.2. Gold Side

**Figure 4:**
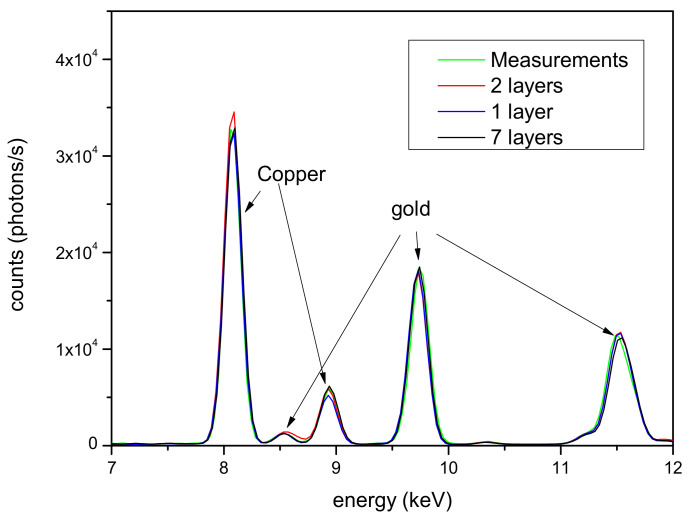
Thigh protector—gold area. Comparison of different models for the sample structure.

**Figure 5:**
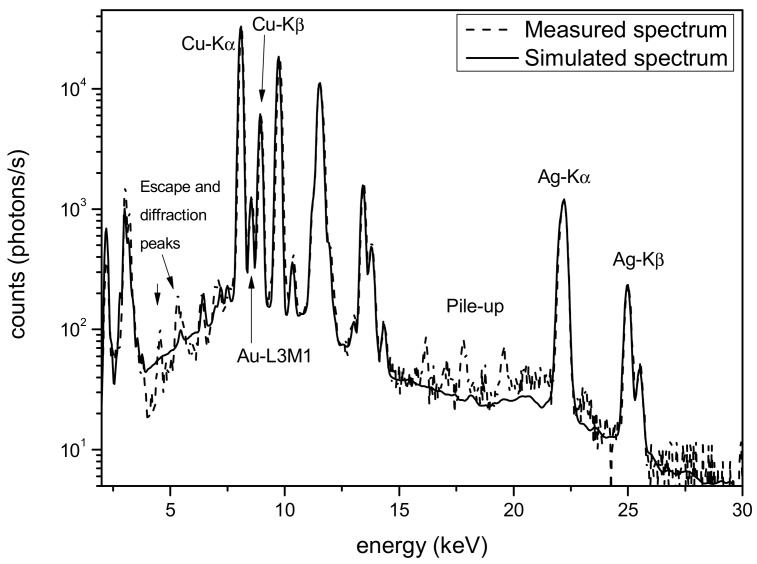
Thigh protector—gold area. Comparison of measured spectrum (dashed line) and simulated spectrum (solid line). The peaks between 4 and 6 keV and those around 16 keV are due to escape, diffraction and pile-up phenomena, respectively, which is why they were not considered when creating the fit.

Even the gold side of the artifacts was simulated according to different models, ranging from a single layer to seven layers. In Figure 4, a comparison of some of these configurations is presented. Only the part of the spectra where the differences are more significant (7–12 keV), corresponding to the copper and gold peaks, is reported, although smaller differences are also observed for the silver peaks. The Tumbaga seven-layer model performs better than others when compared to the experimental spectrum, and both are shown in Figure 5. Even in this clearly different case, performance similar to that observed with the silver alloy is noticed, confirming the capability of the Monte Carlo simulation to discriminate Tumbaga (whether it be silver or gold-based) from simpler multilayer structures.

**Figure 6:**
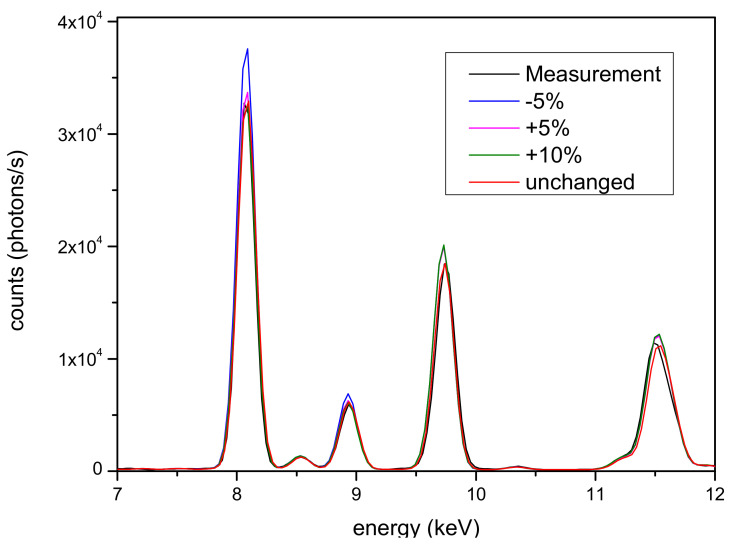
Thigh protector—gold area: Tumbaga model (seven layers) with different slopes of the gradient, expressed a percentage of the change with respect to the best, unchanged slope.

**Figure 7:**
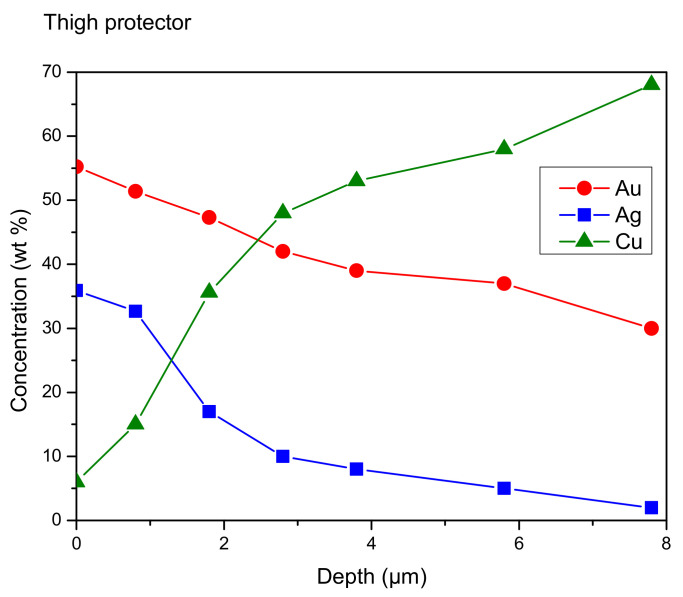
Thigh protector—gold area. Concentration gradient of gold (Au), silver (Ag) and copper (Cu) as a function of depth. You can see the phenomenon of surface enrichment of the gold, compared to the innermost areas of the alloy.

The influence of different slopes of the gradient was also studied, and the results are shown in Figure 6. Differences of as little as 5% are easily detected. In Figure 7 and Table 2, the best gradient, and layer-by-layer concentration, are reported.

**Figure 8:**
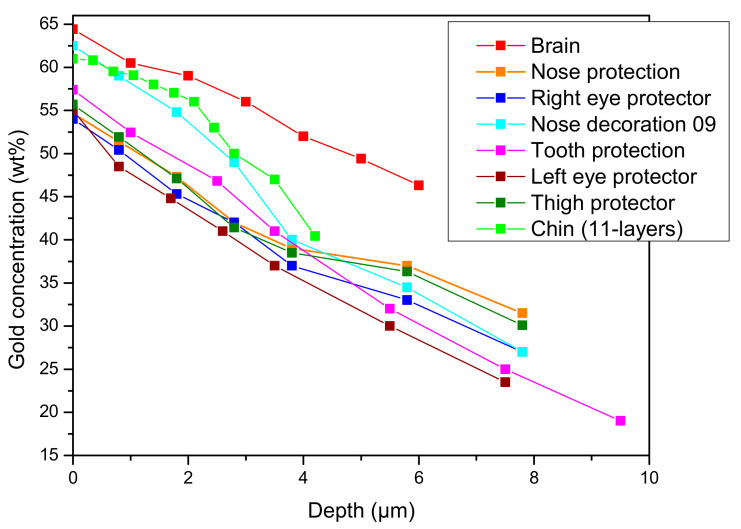
Gold profile of gold concentration of Tumbaga alloys from the same burial. The name is the same as in ref. [4]. The symbols indicate the relative thickness of each layer composing the gradient profile.

It is of interest to establish the minimum change in the concentration detectable in each layer. In order to determine this, the first upper layer and the last one were considered, and the gold content was changed one layer at a time. As expected, the minimum changes in the latter inner layer are higher than in the first layer. A minimum variation of 1% in the gold content is detected for the inner layer. This value decreases to 0.02% for the first layer, which is considerably lower than the corresponding statistical Poissonian error in the measured spectrum, intrinsic to the interaction phenomena. This confirms the high sensitivity of this approach. Based on this fact, it seems of interest to compare the Tumbaga profile of the sample examined here with others published in a previous paper [4]. The results are summarized in Figure 8. The gradient profiles can be grouped into two sets: one composed of the Brain, Chin, Tooth protection, Eye protectors, Nose decoration 09 and Nose protection samples (see ref. [4] for the description and pictures), while the other forms a separate set. Clearly, all the samples represented in Figure 8 are similar and all are Tumbaga. However, while the gradient profile is similar, some of them exhibit a higher surface concentration of gold (Brain, Nose decoration09 and Chin), indicating better depletion action. In one case, the Brain sample, this is due to the different alloy containing more gold.

This type of analysis, conducted for the first time to the best of our knowledge, will provide a new tool for the study of this kind of metallurgy.

## 4. Conclusions

In this paper, a unique bimetallic artifact manufactured by the Moche civilization was studied. Its significance lies not only in its uniqueness, but also in its contribution to the development of metallurgy in Central America. The sample, composed of two sides, was analyzed non-destructively using XRF spectroscopy coupled with Monte Carlo simulations. This approach has once again demonstrated its effectiveness in the analysis of complex multilayered structures such as Tumbaga gold, where it is impossible to sample the artifacts or move them outside the Museum. For the first time, the sensitivity of Monte Carlo simulation of the Tumbaga profile to gold concentration changes in just one layer was tested, revealing that variations of concentration as low as 1% are detectable in the inner layer, thus confirming the quality and sensitiveness of this approach. Furthermore, for the first time, to the best of our knowledge, a comparison with other Tumbaga objects from the same burial site was conducted to observe differences in the profiles of gold concentration between the samples. It was possible to group all the objects analyzed here and in a previous work into two sets, where the larger set shows the same composition of the alloy and even the same kind of gold concentration profile, thus proving a certain degree of standardization in the manufacturing technique. This, in principle, paves the way to the recognition of the artisan and, consequently, the provenience of these artifacts.

## Figures and Tables

**Table 1:** Double-layer silver alloy structure. The first layer is the corrosion (patina) layer. All the values are expressed as percentages (%). Chemical elements with a concentration lower than 0.1% are not reported.

Layer No.#	S	Ca	Cr	Cu	Zn	Ag	Au	Pb
1	0.88	0.45	0.1	9.5	-	14.4	0.85	-
2	-	-	-	30.0	0.2	66.2	3.4	0.2

**Table 2:** Seven-layer gold alloy structure. All the values are expressed as percentages (%).

Layer No.	Cr	Fe	Ni	Cu	Ag	Au
1	0.1	0.21	0.22	4.0	35.9	55.8
2	-	0.1	-	14.0	32.7	53.25
3	-	-	-	34.6	17.0	48.3
4	-	-	-	47.0	11.0	42.0
5	-	-	-	53.0	8.0	39.0
6	-	-	-	57.0	6.0	37.0
7	-	-	-	67.0	3.0	30.0

## Data Availability

All the data used in this paper will be made available upon request.
